# High levels of toxigenic *Clostridioides difficile* contamination of hospital environments: a hidden threat in hospital-acquired infections in Kenya

**DOI:** 10.1099/acmi.0.000171

**Published:** 2020-09-18

**Authors:** Erick Odoyo, Cecilia Kyanya, Winnie Mutai, Lillian Musila

**Affiliations:** ^1^​ United States Army Medical Research Directorate – Africa, PO Box 606-00621, Nairobi, Kenya; ^2^​ University of Nairobi, PO Box 30197-00200, Nairobi, Kenya

**Keywords:** hospital-acquired infections, hospital environments, toxigenic *Clostridioides difficile*

## Abstract

**Introduction:**

The contribution of *Clostridioides difficile* (formerly *Clostridium difficile *) to the burden of hospital-associated infections (HAIs) remains undetermined in many African countries.

**Aim:**

This study aimed to identify a sensitive and readily adaptable *C. difficile* detection assay and to evaluate the *C. difficile* HAI risk in Kenya.

**Methodology:**

Sterile swabs in neutralizing buffer were used to sample equipment or surfaces that patients and clinical staff touched frequently. These swabs were either plated directly on chromogenic agar or cultured in an enrichment broth before plating. The swab suspensions, enrichment broth and plate cultures were screened by quantitative PCR (qPCR) to determine the most efficient detection method. The HAI risk was evaluated by testing the *C. difficile *-positive samples by qPCR for the A, B and binary toxins.

**Results:**

*
C. difficile* was detected on 4/57 (7.0 %) equipment and surfaces by direct culture. The additional enrichment step increased the detection rate 10-fold to 43/57 (75.4 %). In total, 51/57 (89.5 %) environmental samples were positive for *C. difficile* detected through either culture or qPCR. The genes encoding the primary toxins, *tcdA* and *tcdB*, were detected on six surfaces, while the genes encoding the binary toxins, *cdtA* and *cdtB*, were detected on 2/57 (3.5 %) and 3/57 (5.3 %) surfaces, respectively. Different *C. difficile* toxin gene profiles were detected: the *tcdA+/tcdB*− gene profile on 4/10 (40 %) high-touch surfaces, *tcdA−/tcdB*+ on 3/10 (30 %) surfaces, *tcdA+/tcdB+/cdtA+/cdtB+* on 2/10 (20 %) surfaces and *tcdA−/tcdB+/cdtB+* on one high-touch surface.

**Conclusion:**

The widespread contamination of hospital environments by toxigenic *C. difficile* gives a strong indication of the high risk of *C. difficile* infections (CDIs). The two-step culture process described can easily be adapted for monitoring hospital environment contamination by *C. difficile *.

## Introduction

*Clostridioides difficile* (formerly *Clostridium difficile *) is a Gram-positive, anaerobic, spore-forming bacterium that can survive for extended periods on environmental healthcare surfaces [1]. *C. difficile* is a significant cause of hospital-acquired infections (HAIs) in the developed world [2, 3]. Toxigenic forms of *C. difficile* cause mild to severe antibiotic-induced diarrhoea with symptoms ranging from mild to severe diarrhoea and haemorrhagic colitis [4]. In contrast to the situation in developed countries, *C. difficile* epidemiology in developing countries, and Africa in particular, remains poorly understood. The burden of *C. difficile* infections (CDIs) in these countries may be higher than appreciated because of evidence of risk factors such as inappropriate use of antibiotics, including high levels of antimicrobial self-medication [5]. There are also many cases of diarrhoea in Africa whose aetiologies remain unknown [6], in part because many studies carried out in the continent do not investigate all causes of diarrhoeal diseases, including *C. difficile* [7]. These studies are likely limited by the strict anaerobic nature of *C. difficile* requiring specialized media and anaerobic conditions to culture the bacterium, which are often not available in resource-limited settings. Two toxins, TcdA and TcdB, are the major virulence factors in *C. difficile* [4] and are detected either using antibody-based or cytotoxicity assays. These toxins, encoded by the genes *tcdA* and *tcdB*, form the basis of nucleic acid amplification tests for the identification of toxigenic *C. difficile *. In addition to the primary toxins, some *C. difficile* strains produce binary toxins, CDTa and CDTb, encoded by genes *cdtA* and *cdtB*, respectively [4]. Strains producing these binary toxins have, in some studies, been associated with increased severity and mortality [8].

*C. difficile* prevalence rates ranging from 0–93.3 % of patients presenting with diarrhoea have been reported in Africa [9–16]. This considerable variation can be accounted for in part by the differences in laboratory detection procedures [17], which, in some instances, have been suboptimal [10]. Indeed, disparities in laboratory testing practices are prevalent even in developed countries with greater resources [2], highlighting the need for standardized detection methods, particularly in Africa, where healthcare-associated infections are disproportionately high compared to developed countries [18].

Hospital environments act as a reservoir for *C. difficile* and have previously been linked to the acquisition of CDIs [1, 19, 20]. *C. difficile* can persist in the environment for up to 5 months as spores [1] that are resistant to commonly used disinfectants and alcohol-based hand hygiene products, thus posing a public health risk [21, 22]. It is, therefore, essential to carry out microbial monitoring of the hospital environment to determine the presence of toxigenic *C. difficile* to prevent CDIs [23]. This study aimed to identify a sensitive and readily adaptable *C. difficile* detection assay and to evaluate the *C. difficile* HAI risk in the Kisii Teaching and Referral Hospital (KSI) in Kenya.

## Methods

This descriptive laboratory-based study was conducted at KSI. KSI is a large government referral hospital with a 450-bed capacity that offers a range of services to patients living mostly in western Kenya. KSI is also part of a network of hospitals involved in human and environmental surveillance of key HAI pathogens, including *C. difficile *, carried out by the United States Army Medical Research Directorate – Africa. Four KSI hospital departments were sampled: paediatric, newborn, surgical and outpatient. These areas were selected based on the hospital’s identification of departments with high frequencies of HAIs. Specific equipment or surfaces that patients and clinical staff frequently touched or handled, known as high-touch areas, were identified for swabbing within each of the four hospital departments. All swabs were collected on a single day (3 September 2018).

### Sampling and laboratory processing procedures

Sterile swabs in neutralizing buffer (NB) (ESK sampling kit, Puritan, Guildford, ME, USA) were used to sample 57 high-touch surfaces and pieces of equipment. The sampled high-touch surfaces included: 13 intravenous i.v. poles, 12 sets of patient bedding, 11 bed rails, 5 doorknobs, 4 newborn incubators, 4 bed trays, 3 room sinks, 2 suction tubes, an oxygen concentrator, a nurses’ desk and a monitor control panel. The neutralization buffer maintains the viability of the bacteria on the swab sample during transportation [23] and neutralizes the effects of residual surface disinfectants [24], which could increase the number of false-negative samples. The swabs were rotated with firm pressure over a 500 cm² target area and then this was repeated at perpendicular angles for maximum recovery. For flat surfaces, a template was used to define the swabbed area; otherwise, for smaller objects/surfaces, the whole of the target area was swabbed. The swabs were then shipped at 4 °C to the testing laboratory at the Kenya Medical Research Institute (KEMRI), Nairobi, and received for processing within 24 h.

### Isolation and detection of *C. difficile*

Each swab was vortexed in the NB buffer and then cultured using two different methods. In the first method, 100 µl of the sample was plated directly in chromogenic agar (CHROMagar *C. difficile,* Paris, France) and incubated in an anaerobic jar (Oxoid, Waltham, MA, USA) with an AnaeroGen system (Oxoid, Waltham, MA, USA) at 37 °C for 48 h. In the second method, 100 µl of the NB buffer suspension was enriched in a fastidious anaerobe broth, (FAB) (Lab M, Bury, UK) for 48 h. The FAB medium contains the reducing agents, sodium thioglycollate and l-cysteine, which provide optimal growth conditions for the recovery of fastidious anaerobes such as *C. difficile* [25]. The liquid culture was then plated on chromogenic agar and incubated for 48 h. Chromogenic agars limit the growth of contaminating bacteria often present in environmental specimens and provide visual indications of the presence of *C. difficile *, thereby facilitating the detection of *C. difficile* in culture [26]. *C. difficile* isolates were identified on the culture plate by their flat, medium-sized, colourless colonies with ground glass appearance and the characteristic horse dung odour. On the chromogenic agar, the colonies fluoresced under long-wave UV illumination (365 nm) [27].

Bacterial DNA was extracted using the *Quick*-DNA miniprep kit (Zymo Research, Irvine, CA, USA) according to the manufacturer’s instruction from the swab suspension in NB buffer, the FAB enrichment broth culture and the colonies on the culture plates as described in the culture procedures above. The *C. difficile* strain DSM 27147 (Leibniz Institute DSMZ, Brunswick, Germany) was used as a control for the culture and quantitative PCR (qPCR) detection of *C. difficile *. To confirm the identity of the isolated *C. difficile *, qPCR targeting the *C. difficile *-specific triosephosphate isomerase (*tpi*) gene was performed using published primers [28]. The samples confirmed to have *C. difficile* were then screened in a separate qPCR reaction using published primers specific for genes encoding the primary toxins, *tcdA* and *tcdB*, and the binary toxins, *cdtA* and *cdtB* [29]. The 20 µl PCR reaction consisted of 10 µl Luna Universal qPCR Master Mix (New England Biolabs, Ipswich, MA, USA), 2 µl of extracted DNA, 1 µl of 10 pM primer and 7 µl of nuclease-free water. The qPCR reactions were carried out in a Mic qPCR thermocycler (Bio Molecular Systems, Upper Coomera, QLD, Australia) using the cycling parameters: initial denaturation at 95 °C for 60 s followed by 40 cycles of 95 °C for 15 s, 55 °C for 30 s and 72 °C for 30 s. A final SYBR green dye dissociation step from 72 to 95 °C at 0.3 °C s^−1^ was used to determine the melting temperatures of the amplicons. The specificity of the qPCR reactions was determined by observing peaks at 79 °C (for *tcdA* genes) and 75 °C (for *tcdB* genes) on the SYBR green dye dissociation curves and band sizes of 369 (for *tcdA* genes) and 160 bp (for *tcdB* genes) by agarose gel electrophoresis.

## Results

### Detection of *C. difficile*

Of the 57 equipment and hospital surfaces sampled, *C. difficile* was detected on 4/57 surfaces (7.0 %) by direct plating of the swab on the chromogenic agar. Enrichment of the specimen in FAB followed by plating on chromogenic agar increased detection rates 10-fold to 43/57 (75.4 %). The qPCR detection rate following enrichment and plating on chromogenic agar was also 43/57 (75.4 %). The qPCR detection rates for *C. difficile* in the NB buffer and the FAB enrichment broth were 28/57 (49.1 %) and 19/57 (33.3 %), respectively. Overall, 51/57 (89.5 %) of the sampled high-touch surfaces were contaminated with *C. difficile* detected either by culture or by qPCR.

### Surfaces and equipment contaminated with *C. difficile*

The items most contaminated with *C. difficile* were patient beds (12/13), i.v. poles (11/13), room inner doorknobs (5/5), newborn incubators (4/4) and room sinks (3/3) (Table 1). All items sampled in the outpatient, surgical and newborn departments were contaminated with *C. difficile *. Seven of the 13 (53.8%) items tested in the paediatric department were contaminated with *C. difficile. C. difficile* toxin genes were detected from 10/51 (19.6 %) of the *C. difficile-positive* hospital surfaces. These surfaces included the paediatric, newborn, outpatient and surgical department doorknobs, i.v. poles, patient beds, baby incubators and sinks (Table 1).

**Table 1.** The items screened for *C. difficile* within each department, indicating widespread bio-contamination in the hospital

Hospital department	Item(s)	No. of items contaminated with *C. difficile */no. of items sampled	No. of items contaminated with toxigenic *C. difficile */no. of items sampled
Surgical	Patient bedding	5/5	0/5
	Bed rails	5/5	1/5
	i.v. poles	5/5	0/5
	Room sinks	2/2	0/2
Paediatric	Patient bedding	2/3	1/3
	Bed rails	2/3	0/3
	Bed trays	1/2	0/2
	i.v. poles	0/2	0/2
	Suction tubes	1/2	0/2
	Doorknob	1/1	1/1
Newborn	Newborn incubators	4/4	2/4
	i.v. poles	3/3	1/3
	Bed tray	1/1	0/1
	Doorknob	1/1	0/1
	Room sink	1/1	1/1
Outpatient	Patient bedding	4/4	1/4
	Bed rails	3/3	0/3
	i.v. poles	3/3	1/3
	Nurses’ desk	1/1	0/1
	Doorknobs	3/3	1/3
	Oxygen concentrator	1/1	0/1
	Monitor control panels	1/1	0/1
	Bed tray	1/1	0/1
**Total**		**51/57 (89.5 %)**	**10/57 (17.5 %)**

### Toxin gene detection from the high-touch surfaces

The genes encoding the primary toxins, *tcdA* and *tcdB*, were detected on six surfaces, while the genes encoding the binary toxins, *cdtA* and *cdtB*, were detected on 2/57 (3.5 %) and 3/57 (5.3 %) surfaces, respectively. Different *C. difficile* toxin gene combinations were detected: *tcdA+/tcdB*− on 4/10 surfaces, *tcdA−/tcdB*+ on 3/10 surfaces, *tcdA+/tcdB+/cdtA+/cdtB+* on 2/10 surfaces and *tcdA−/tcdB+/cdtB+* on one surface (Table 2).

**Table 2.** The toxin gene profiles detected from the swabbed surfaces

Toxin gene combinations detected from the swabbed surfaces	No. of contaminated surfaces/no. of swabbed surfaces (percentage)
*tcdA+/tcdB−/cdtA−/cdtB−*	4/57 (7.0)
*tcdA−/tcdB+/cdtA−/cdtB−*	3/57 (5.3)
*tcdA+/tcdB+/cdtA+/cdtB+*	2/57 (3.5)
*tcdA−/tcdB+/cdtB+*	1/57 (1.8)

## Discussion

The detection of *C. difficile* is hindered by several factors, including the poor selectivity of the media used for isolation [26], the requirement for anaerobic conditions for culture, which remain unavailable in most laboratories in developing countries, as well as what is referred to as ‘a low clinical suspicion index’ among clinicians for CDIs [30]. In this study, the combination of the enrichment in FAB and culture on chromogenic agar proved to be a rapid and easy method for the detection of *C. difficile *. All the isolates presumptively identified as *C. difficile* on the chromogenic agar were confirmed by gene-specific PCR to be *C. difficile,* indicating the high specificity of this method. The *C. difficile* chromogenic agar is available in a dehydrated form with a long shelf life, making it feasible for storage and prolonged use for both clinical diagnosis and environmental bio-monitoring. Bell jar anaerobic culture proved to be adequate for the creation of a low-throughput anaerobic chamber suitable for low-resource laboratories. However, the study methods employed could not discriminate between *C. difficile* vegetative forms and its spores.

In this study, the recovery rate for *C. difficile* by direct plating was 10-fold lower compared to when an enrichment step preceding plating in the chromogenic agar was applied. The low recovery on the primary plate can be explained by the small surface area swabbed in the study, with plating of only the swab suspension resulting in few spores in the cultured sample, combined with the challenge of culturing spores on solid versus liquid media. The swab suspensions were also not spun down before culture to ensure the capture and concentration of spores, possibly contributing to the low recovery on the primary plate. In other studies, a broth enrichment step led to the optimal recovery of *C. difficile* from hospital surfaces [31–33], indicating that, for environmental samples, the two-step process is necessary to detect the true bioburden of *C. difficile* in the hospital environment. This method can be quickly adapted for *C. difficile* bio-monitoring programmes in hospitals.

Although *C. difficile* strains encoding either TcdA or TcdB toxins have been shown to cause fulminant colitis [4], recent evidence has demonstrated that TcdB is 100–10 000 times more potent than TcdA in several cell types [34, 35]. In the current study, *C. difficile* strains encoding the *tcdB* gene were found in half of the samples containing toxigenic strains, reflecting a high risk of CDI with severe clinical manifestations. Studies carried out in patients with diarrhoea in the African continent have also found a high proportion of the *tcdB+* strains [14, 15, 36]. *C. difficile tcdB+* strains were identified in patients with diarrhoea in the same hospital [37], indicating that these strains play a crucial role in CDIs. *C. difficile* binary toxins confer adenosine diphosphate ribosyltransferase activity [38] and in some studies have been associated with hypervirulent strains that cause increased severity and mortality [8]. However, the specific role of these binary toxins in these adverse outcomes remains to be established. In the current study, both binary genes were detected from high-touch surfaces. Detection of the *cdtB* gene from one surface presents the additional risk of infection with potentially more virulent strains of *C. difficile *. Overall, the high prevalence of toxin genes of *C. difficile* in the hospital environment in the current study indicates a significant risk of HAI.

Hospital surface contamination with *C. difficile* has previously been linked to the acquisition of CDIs [1, 19, 20, 39]. The current study found widespread *C. difficile* contamination in the study hospital environment. High-touch surfaces such as patient beds, room doorknobs and newborn incubators were contaminated with toxigenic *C. difficile *. i.v. poles, which are highly portable from one department to another and used between patients, were highly contaminated and were identified as a critical fomite for the transmission of nosocomial pathogens. Contaminated room sink taps represent an additional risk for transmission of *C. difficile* in the study hospital. If the decontamination of sink areas is not adequate, it could act as a significant reservoir for CDI because handwashing is the primary infection prevention tool.

*C. difficile* contamination is frequently found in rooms previously housing CDI patients [1, 33, 40]. The widespread *C. difficile* contamination reported here suggests that *C. difficile* could be a significant cause of diarrhoea in the study hospital. The high levels of CDIs detected in patients with diarrhoea at the same hospital in a recent study [16, 37] support this assertion. With the ease of access to and inappropriate use of antibiotics in the study region [5], which could trigger CDI, *C. difficile* could well be a problem of greater magnitude than previously recognized. This study is the first to examine *C. difficile* in a hospital environment in Kenya and identify environmental reservoirs that could be important transmission factors. Sequencing of these environmental strains and comparison with clinical isolates from the same hospital setting will provide more information about HAIs. The widespread contamination reported in this study also points to low adherence to infection prevention and control practices in the study hospital. With the emerging evidence of *C. difficile* resistance to chlorine-releasing agents [41], strategies that can be applied to prevent hospital-acquired CDIs include: contact precautions, mechanical removal of spores where possible, and hospital staff and patient education, as well as robust antibiotic stewardship programmes [22, 42].

## Conclusion

Routine laboratory investigations that seek the aetiological agents of diarrhoea in Kenya often do not include *C. difficile* because of the prevailing thought that it is not a significant cause of diarrhoeal disease in the region. The extensive contamination of hospital environments and the toxin genes of the detected *C. difficile* described in this study give a strong indication of the high risk of CDI with severe clinical manifestations in the study hospital and possibly other hospitals in the country. The two-step culture described in this study can be used to detect *C. difficile* from hospital environments to monitor the effectiveness of implemented cleaning practices appropriate for *C. difficile *. Continued and more extensive bio-monitoring of hospitals will be necessary to continue to expose and address the threat of CDI in Kenyan hospitals and the community.
